# Characterization of Critical Functions of Long Non-Coding RNAs and mRNAs in Rhabdomyosarcoma Cells and Mouse Skeletal Muscle Infected by *Enterovirus* 71 Using RNA-Seq

**DOI:** 10.3390/v10100556

**Published:** 2018-10-11

**Authors:** Ying Li, Chao Zhang, Luwei Qin, Dong Li, Guangyuan Zhou, Dejian Dang, Shuaiyin Chen, Tiantian Sun, Rongguang Zhang, Weidong Wu, Yuanlin Xi, Yuefei Jin, Guangcai Duan

**Affiliations:** 1 Department of Epidemiology, College of Public Health, Zhengzhou University, Zhengzhou 450001, China; lyshadow2016@163.com (Y.L.); cc954957400@163.com (C.Z.); lidongstu@163.com (D.L.); 15225134317@163.com (D.D.); sychen@zzu.edu.cn (S.C.); suntiantian2022@163.com (T.S.); zrg@zzu.edu.cn (R.Z.); xyl@zzu.edu.cn (Y.X.); 2 School of Public Health, Xinxiang Medical University, Xinxiang 453003, China; qinlwxx@163.com (L.Q.); 18770227768@163.com (G.Z.); wdwu2013@126.com (W.W.)

**Keywords:** *Enterovirus* 71, hand-foot-mouth disease, long non-coding RNA, RNA sequencing, RD cells, skeletal muscle

## Abstract

*Enterovirus* 71 (EV71) is the main pathogen of severe hand-foot-mouth disease (HFMD). Long non-coding RNAs (lncRNAs) are recognized as pivotal factors during the pathogenesis of viral infection. However, the critical functions of lncRNAs in EV71–host interactions have not been characterized. Here, for the first time, we performed global transcriptome analysis of lncRNA and mRNA expression profiles in EV71-infected human rhabdomyosarcoma (RD) cells and skeletal muscle of mice using second-generation sequencing. In our study, a total of 3801 novel lncRNAs were identified. In addition, 23 lncRNAs and 372 mRNAs exhibited remarkable differences in expression levels between infected and uninfected RD cells, while 104 lncRNAs and 2647 mRNAs were differentially expressed in infected skeletal muscle from neonatal mice. Comprehensive bioinformatics analysis included target gene prediction, lncRNA-mRNA co-expression network construction, as well as gene ontology (GO) and Kyoto Encyclopedia of Genes and Genomes (KEGG) pathway analysis mainly focused on differentially-expressed genes (DEGs). Our results suggest that lncRNAs may participate in EV71 infection-induced pathogenesis through regulating immune responses, protein binding, cellular component biogenesis and metabolism. The present study provides novel insights into the functions of lncRNAs and the possible pathogenic mechanism following EV71 infection.

## 1. Introduction

*Enterovirus 71* (EV71) is a member of the *Picornaviridae* family with a single positive-stranded RNA genome [1], and is well known as the main pathogen of hand-foot-mouth disease (HFMD) that occurs mainly in infants and young children [2]. HFMD usually follows a mild and self-limiting course, and the main clinical symptoms are fever, oral vesicles and rashes on the hands, feet and buttocks. However, as a type of neurotropic virus, EV71 infection often results in fatal neurological disorders such as aseptic meningitis, acute encephalitis, cardiopulmonary failure and polio-like acute flaccid paralysis [3]. In recent years, the outbreaks of EV71 infection have become a serious threat to the health of children across the Asia–Pacific region [4]. Therefore, it is extremely urgent to find out the pathways of EV71 pathogenesis.

Long non-coding RNAs (lncRNAs) are emerging as a large class of non-coding RNAs (ncRNAs) whose transcripts are at least 200 nucleotides long [5]. Based on genomic region of origin and proximity to their relative protein-coding genes, lncRNAs are classified into several categories including exon sense-overlapping, intron sense-overlapping, natural anti-sense, intron anti-sense, bidirectional, and intergenic lncRNAs [6]. LncRNAs lack the capacity to encode proteins and the vast majority of lncRNAs are regarded asgenomic ‘noise’ [7]. However, in the past decade, the overwhelming development of transcriptomics technologies suggested that lncRNAs could modulate gene expression as enhancers, co-activators, co-repressors, decoys or scaffolds, either through *cis*-regulatory or *trans*-regulatory interactions with DNA, RNA, or protein molecules in various physiological and pathological processes [8,9].

With the prevalent application of next-generation high throughput sequencing techniques and bioinformatics analyses, accumulating evidence indicates that lncRNAs serve as a crucial mediators involved with regulation of chromatin structure, cell cycle and differentiation, formation of organs and tissues, reproduction, senescence, as well as in disease development [10,11,12,13,14]. Recent studies have revealed the differential expression of host lncRNAs in response to viral infection and their potential roles in modulating host innate immunity and inflammatory reactions [15]. However, the role of lncRNAs in EV71 infection is still unknown.

Herein, we find out the differential expression profiles of lncRNAs and mRNAs in human rhabdomyosarcoma (RD) cells and mice skeletal muscle after EV71 infection. Systematic bioinformatics analysis utilizing GO (gene ontology), the Kyoto Encyclopedia of Genes and Genomes (KEGG) pathway, and lncRNA-mRNA co-expression networks were performed to define the potential role of lncRNAs in EV71–host interactions.

## 2. Materials and Methods

### 2.1. Ethics Statement

The number of animals and procedures used in the present study were reviewed and approved by the Life Sciences Institutional Review Board of Zhengzhou University on 1 September 2017 (project identification code: 201709-006). The experiments were performed strictly in accordance with the Guidelines of Zhengzhou University for Animal Experiments.

### 2.2. EV71 Virus and Cell Treatment

As described previously, EV71 strain (ZZ1350) was isolated from a non-fatal case with central nervous system (CNS) involvement in the Children’s Hospital of Zhengzhou (Zhengzhou, Henan, China) [16]. RD cells were purchased from the Chinese Academy of Sciences Cell Bank (Shanghai, China) and cultured in Dulbecco’s Modified Eagle Medium (DMEM, Gibco Company, New York, NY, USA) supplemented with 10% fetal bovine serum (FBS) (Gibco Company, New York, NY, USA). Median tissue culture infective dose (TCID_50_) were determined by plaque assay using RD cells. Working stocks (1 × 10^8^ PFU per mL) were stored at −80 °C. When RD cells grew to 90% confluency in 6-well plates, cultures were infected at multiplicity of infection (MOI) of 1. EV71 virus was added in 3 wells, and the same volume of culture medium was added to another 3 wells as blank controls. The cytopathic effect (CPE) in RD cells was evaluated by imaging under light microscopy (original amplification: 100×) and confocal microscopy (original amplification: 400×) at 24 h post-infection (hpi).

### 2.3. Animals

Specific pathogen free 3-day-old Balb/c mice were obtained from the Medical Animal Center in Zhengzhou University (Zhengzhou, Henan, China) and maintained in an individual ventilated cage (IVC) system. Six Balb/c mice were intraperitoneally (i.p.) inoculated with EV71 (2 × 10^6^ PFU) or the same volume of saline (n=3 for each group). At 7 days post infection (dpi), the mice were euthanized by CO_2_ inhalation and the skeletal muscle was separated and immediately homogenized for further RNA isolation. Histopathological examinations of skeletal muscle were evaluated by haematoxylin and eosin (H&E) staining, and viral VP1 protein expression in tissue was examined by immunohistochemical (IHC) staining as previously described [17].

### 2.4. RNA Isolation

Total RNA from human RD cells and mice skeletal muscle was isolated using Trizol reagent (Invitrogen Corporation, Carlsbad, CA, USA) according to the manufacturer’s guidelines. RNA quality was evaluated on 1% agarose gels, and the concentration and purity were determined by measuring the absorption at 260/280 nm using a NanoDrop ND-2000 (Thermo Fisher Scientific, Waltham, MA, USA).

### 2.5. RNA Library Construction and Sequencing

RNA (3 μL) was prepared for RNA-seq analysis. Ribosomal RNA was first eliminated using a Ribosomal RNA Removal Kit (Illumina, San Diego, CA, USA) to purify and fragment mRNA. Subsequently, first strand cDNA and second strand cDNA were synthesized using random hexamer primers, followed by end repair, poly-A 3′ ends, ligation of adapters and enrichment of DNA fragments to construct the RNA-seq library with the TruSeq RNA LT Sample Prep Kit (Illumina, USA). Library quality was assessed on the Agilent Bioanalyzer 2100 system (Agilent Technologies, Santa Clara, CA, USA). Next-generation high throughput sequencing of 12 samples was performed on the Illumina Hiseq X Ten platform (150 bp paired-end reads) from Genergy Bio (Genergy Biotechnology Co. Ltd., Shanghai, China).

### 2.6. The Prediction and Classification of New lncRNAs

**Figure 1:**
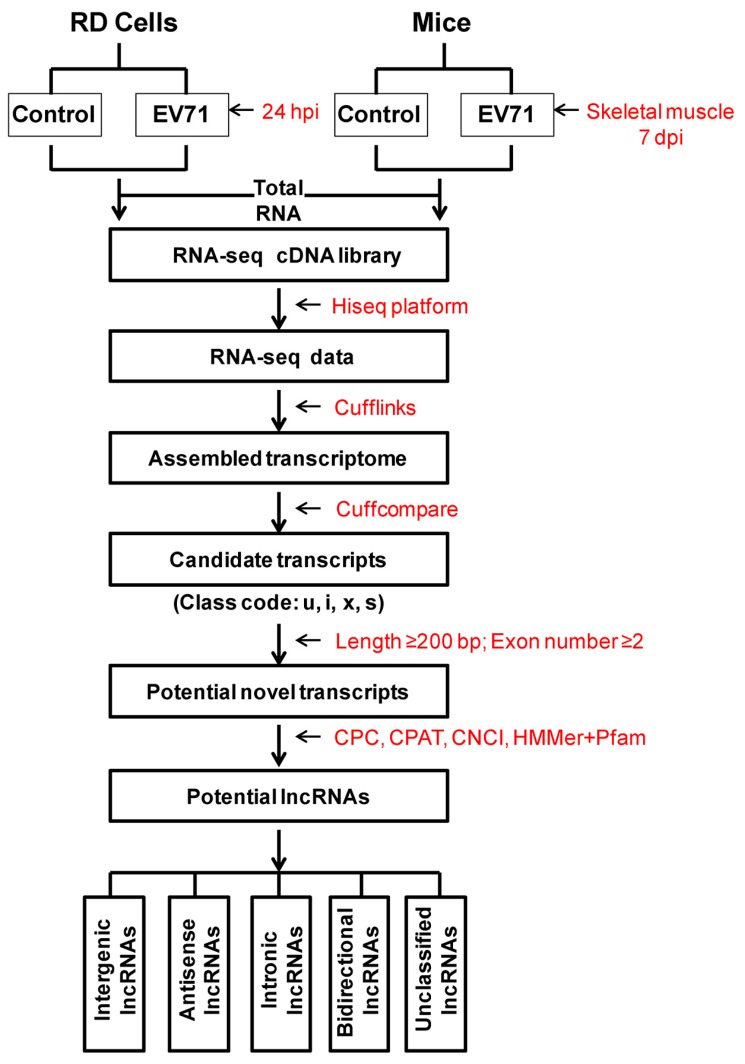
Flow chart for novel lncRNA identification and classification. RD = rhabdomyosarcoma. EV71 = *Enterovirus* 71. hpi = hours post-infection. dpi = days post-infection. lncRNAs = long non-coding RNAs. The CPC, CPAT, CNCI and HMMer+ Pfam are software for predicting the coding ability of transcripts.

As shown in Figure 1, high quality clean data were assembled with Cufflinks v2.2.1, and then we filtered the assembled novel transcripts according to following steps: (1) According to cuffcompare, only transcripts class-coded as “u” (unknown intergenic transcript), “i” (a transfrag falling entirely within a reference intron), “x” (exonic overlap with reference on the opposite strand) and “s” (an intron of the transfrag overlapping a reference intron on the opposite strand) were selected as candidate lncRNAs; (2) To reduce false-positive transcripts, non-transcript sequences caused by the inherent errors in the assembly process need be removed from candidate lncRNAs. Transcripts with lengths greater than 200 bp with at least two exons were retained. (3) The CPC, CPAT, CNCI and HMMer+ Pfam tools were utilized to predict the coding ability of transcripts. The intersection of CPC score<0, CPAT score≤0.364, CNCI score<0 and Pfam showing “noncoding” were the final newly predicted lncRNAs. These lncRNAs were segmented into several categories based on the position to their relative protein-coding genes as described previously [6].

### 2.7. Identification of Differentially-Expressed Genes

To evaluate the expression of transcripts, fragments per kilo-base of transcript per million fragments mapped (FPKM) were calculated by cuffidiff (v2.1.1) based on the length of the fragments and the read counts mapped to those fragments [18]. In general, FPKM≥0.1 demonstrates that transcripts are expressed. According to the design of this experiment, we used the cuffidiff program to screen for differentially-expressed genes (DEGs) between EV71 and negative groups both in RD cells and the skeletal muscle of mice. We considered fold-change (FC)≥2 and p<0.05 as the filter to identify significant DEGs between infected and non-infected samples.

### 2.8. Prediction of Target Genes of Differentially-Expressed lncRNAs

LncRNAs can regulate the expression level of target protein-encoding genes located on adjacent transcripts by *cis*-acting regulation. We defined the target mRNAs 100 kb upstream and downstream away of identified lncRNAs as the threshold. LncRNAs can also regulate the expression level of protein-encoding genes in a *trans* fashion. Depending on the Pearson’s correlation coefficient (PCC) between differentially-expressed lncRNAs and their target mRNAs, we conducted the lncRNA-mRNA co-expression network using Cytoscape software (The Cytoscape Consortium, Oakland, CA, USA) to define the interactions between lncRNAs and mRNAs which are differentially expressed.

### 2.9. GO Annotations and KEGG Enrichment

Gene ontology (GO) provides the functional annotation and classification of molecular functions, biological processes and cellular component aspects of identified differentially-expressed genes (http://www.geneontology.org). The Kyoto Encyclopedia of Genes and Genomes (KEGG) is a database resource for understanding high-level functions for large-scale molecular datasets (http://www.genome.jp/kegg/). These databases were each utilized to understand the role of differentially-expressed target protein-encoding RNAs [19]. Functional analysis was performed using the database for annotation, visualization and integrated discovery (DAVID) v6.8. All genes from *Homo sapiens* and *Mus musculus* were selected as background lists, and the target mRNAs were chosen as candidate lists. Meanwhile, Fisher’s exact test was used to calculate the *p* value according to the annotations, and the rich factor was calculated based on the numbers of symbols in the list.

### 2.10. qPCR Confirmation

Total RNA from the samples were used for RNA-seq. cDNA was synthesized using a PrimeScript RT reagent kit with a gDNA Eraser (TaKaRa, Tokyo, Japan). PCR reactions (20 μL total volume) included 2 μL cDNA product and 10 μL TB Green Premix Ex Taq II I (Tli RNaseH Plus) (TaKaRa, Japan). Reactions were performed with an Applied Biosystems 7500 Real Time PCR System (Applied Biosystems, Waltham, MA, USA), with the following cycle conditions: 95 °C for 30 s, 40 cycles at 95 °C for 5 s and 60 °C for 34 s. β-actin from *Homo sapiens* and *Mus musculus* was chosen as the endogenous reference gene. All the primers used in the present study are listed in Table 1.

### 2.11. Statistical Analysis

SPSS 21.0 (IBM, Chicago, IL, USA) was used for statistical analysis. The experimental data were presented as mean±SEM. Student’s *t*-test was used for estimating the difference in lncRNA expression between EV71 infection groups and control groups in vitro and in vivo. A two-tailed *p* value < 0.05 was considered statistically significant.

## 3. Results

### 3.1. In Vitro and In Vivo Models of EV71 Infection

**Figure 2:**
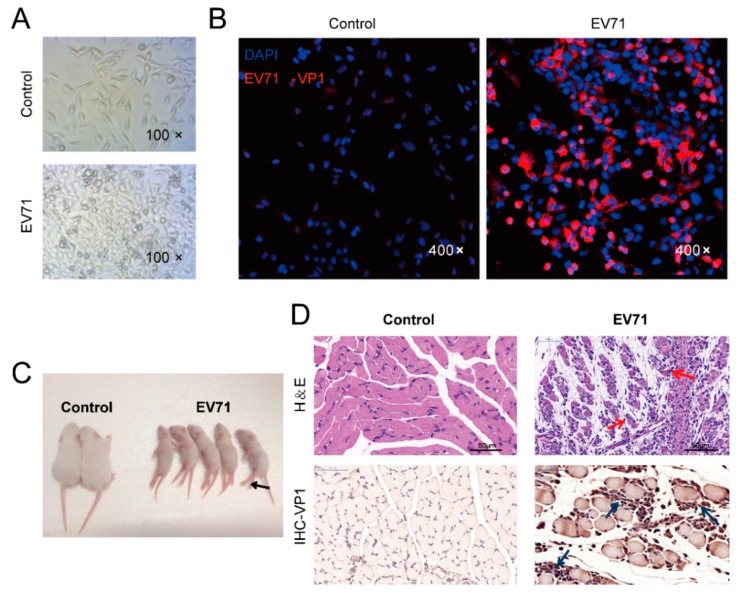
In vitro and in vivo models of EV71 infection. (**A**) Cytopathic effects in RD cells were captured via light microscopy (amplification: 100×) at 24 h post infection. (**B**) EV71 VP1 distribution in RD cells was imaged via confocal microscopy (amplification: 400×) at 24 hpi. (**C**) Clinical presentation in neonatal mice following EV71 infection. (**D**) Histopathological examination and EV71 VP1 distribution (brown) of skeletal muscle in mice at 7 days post infection by haematoxylin and eosin (H&E) staining and immunohistochemistry (IHC) staining, respectively. Red arrows represent inflammatory cell infiltration and necrotizing myositis with rupture of muscle fibers, and blue arrows represent VP1 positive staining area in skeletal muscle of mice. Bar=50 μm. DAPI = 4′, 6-diamidino-2-phenylindole.

As shown in Figure 2A, EV71 infection induced CPE in RD cells at 24 hpi. Figure 2B shows positive expression of EV71 VP1 protein in RD cells at 24 hpi, while no detectable signal was found in control cells. At 7 dpi, the clinical presentations of weight loss and limb paralysis were found in neonatal mice (Figure 2C). Histopathological alterations of mice at 7 dpi were apparent with H&E staining as shown in Figure 2D. EV71-infected skeletal muscle exhibited inflammatory cell infiltration and necrotizing myositis with rupture of muscle fibers. Furthermore, IHC staining showed positive distribution of VP1 in skeletal muscle of mice with EV71 infection at 7 dpi. The results above suggest that RD cells and skeletal muscle of mice were susceptible to EV71, demonstrating that these are valid in vitro and in vivo models to study the mechanisms of EV71 infection.

### 3.2. Identification and Classification of Newly Predicted lncRNAs

**Figure 3:**
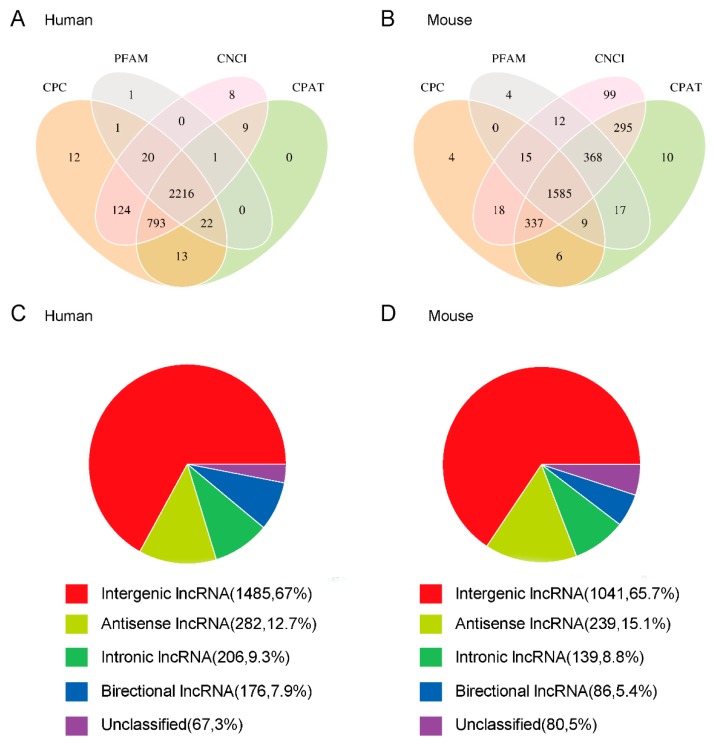
Identification and classification of newly predicted lncRNAs. Venn diagram of the protein-coding ability prediction results in RD cells (**A**) and mice skeletal muscle (**B**). lncRNA classification annotation statistical graph for RD cells (**C**) and skeletal muscle of mice (**D**).

A highly rigorous screening pipeline was developed to identify new lncRNAs in our samples. As shown in Figure 3A,C and Supplementary Table S1, in human RD cells, we obtained 3231 lncRNAs with multi-exon and length≥200 bp from 83,881 primary lncRNAs. Among these, 2216 lncRNAs including 1485 intergenic lncRNAs (67%), 282 anti-sense lncRNAs (12.7%), 206 intronic lncRNAs (9.3%), 176 bidirectional lncRNAs (7.9%) and 67 unclassified lncRNAs (3%) were filtered out with the protein-coding transcripts.

As shown in Figure 3B,D and Supplementary Table S1, 2859 lncRNAs with transcripts length≥200 bp and multi-exon in nature were obtained from 78,519 primary lncRNAs in skeletal muscle of mice. Among these, based on the screening pipeline, 1585 novel lncRNAs including 1041 intergenic lncRNAs (65.7%), 239 anti-sense lncRNAs (15.1%), 139 intronic lncRNAs (8.8%), 86 bidirectional lncRNAs (5.4%) and 80 unclassified lncRNAs (5%) were obtained.

### 3.3. Profiles of Differentially-Expressed lncRNAs and mRNAs

**Figure 4:**
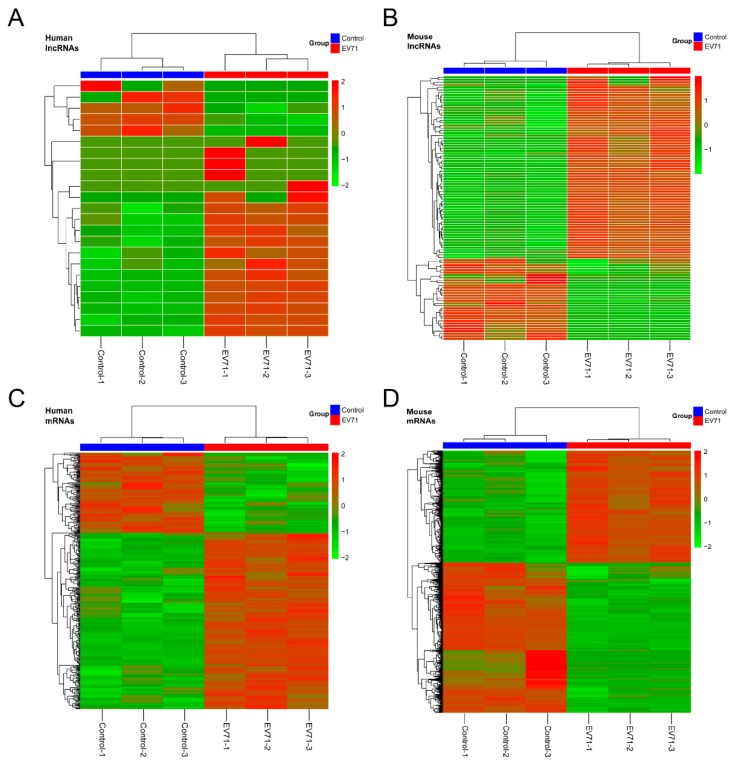
Heat maps of differentially-expressed gene expression ratios (log_2_FC) in EV71-infected human RD cells and skeletal muscle of neonatal mice. Differentially-expressed lncRNAs in RD cells (**A**) and skeletal muscle of mice (**B**) after EV71 infection. Differential expression profiles of mRNAs in RD cells (**C**) and skeletal muscle of mice (**D**). Red and green color denotes high relative expression and low relative expression, respectively.

The expression levels of transcripts were calculated by FPKM using cuffidiff v2.1.1. LncRNAs (24,517), including 2216 newly-predicted lncRNAs, were identified in human RD cells after EV71 infection. The differential expression profiles showed that 23 lncRNAs (18 upregulated and five downregulated) were significantly changed in EV71-infected RD cells. Additionally, 1585 newly predicted lncRNAs of 11,087 lncRNAs were obtained in skeletal muscle of mice. Among these, a total of 104 lncRNAs (72 upregulated and 32 downregulated) were significantly changed. Meanwhile, we found that 256 and 1133 mRNAs were significantly elevated, while 116 and 1514 mRNAs were significantly decreased in EV71-infected human RD cells and mice skeletal muscle, respectively. The general alterations in the expression profiles of lncRNAs and mRNAs were analyzed using hierarchical clustering analysis (Figure 4 and Supplementary Tables S2 and S3). Together, our results suggest that widespread differential regulation of lncRNAs occurs following EV71 infection in vitro and in vivo.

### 3.4. Prediction of Target Protein-Coding RNA

**Figure 5:**
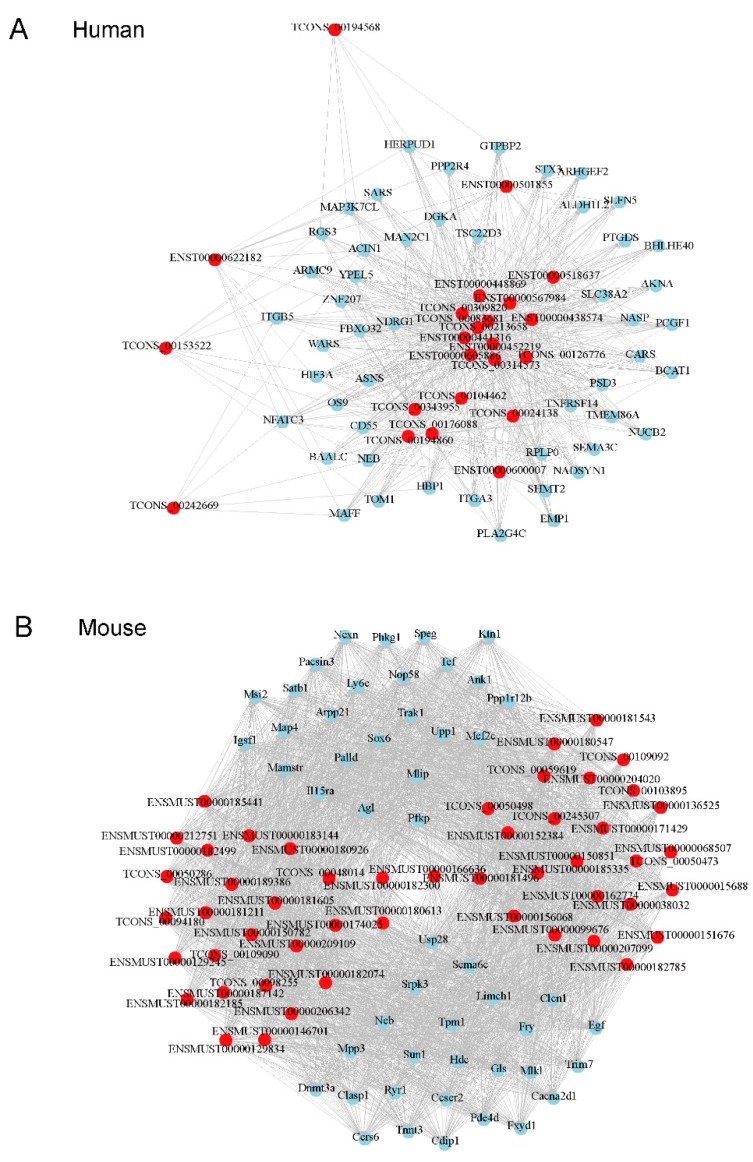
Co-expression network of differentially-expressed lncRNA-mRNA based on Pearson’s correlation coefficient. (**A**) Significant differentially-expressed lncRNAs (23) and the top 50 correlated mRNAs with 685 connection edges in EV71-infected RD cells. (**B**) The top 50 differentially-expressed lncRNAs and their 50 most frequently altered relative mRNAs with 2490 connection edges in EV71-infected skeletal muscle of mice. The red node denotes lncRNA and the surrounding blue node represents mRNA.

To understand the possible functions of lncRNAs, the potential target protein-coding genes in *cis*-and *trans-* regulation was predicted. Our results indicate that 75 protein-coding RNAs could be regulated by differential expression of lncRNAs in RD cells in a *cis* manner, as well as 365 genes in skeletal muscle of mice (Supplementary Table S4). For *trans-*regulation analysis, a lncRNA-mRNA co-expression network was constructed to explore the potential interactions between lncRNAs and mRNAs that were differentially expressed. The lncRNA-mRNA pairs with the strongest correlation (PCC>0.95 or PCC< −0.95, p<0.05) were selected to build the color gradients of a co-expression network (Supplementary Table S5). As shown in Figure 5A, the network of lncRNA-mRNA pairs in EV71-infected RD cells consisted of all differentially-expressed lncRNAs, and the top 50 correlated mRNAs with 685 connection edges. In addition, the top 50 differentially-expressed lncRNAs and relative mRNAs with 2490 connection edges were selected to construct the co-expression of RNA in EV71-infected skeletal muscle of mice (Figure 5B).

### 3.5. GO Annotation and KEGG Pathway Analysis

**Figure 6:**
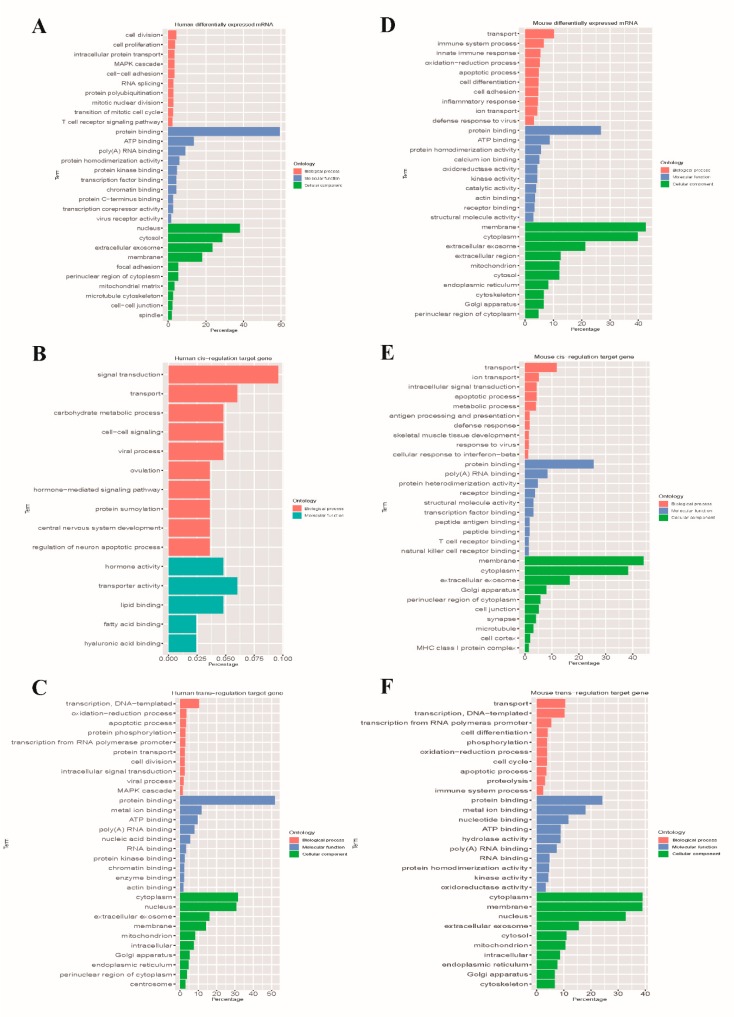
Gene ontology (GO) analysis for differentially-expressed genes upon EV71 infection. (**A**–**C**) The top 10 significantly enriched GO terms in differential expression of mRNA, *cis*- and *trans*-acting target mRNAs in EV71-infected RD cells, respectively. (**D**–**F**) The top 10 significant GO terms in differentially-expressed mRNA, *cis*- and *trans*-regulated genes in EV71-infected skeletal muscle of mice, respectively.

**Figure 7:**
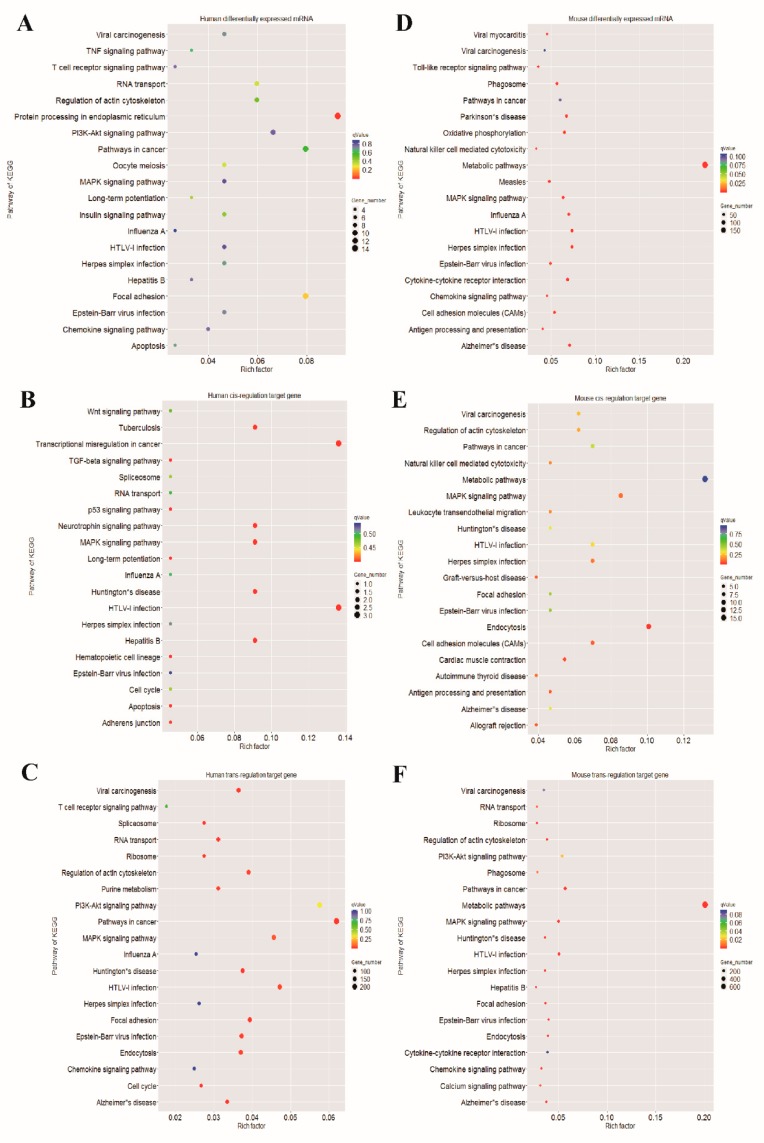
The Kyoto Encyclopedia of Genes and Genomes (KEGG) pathway analysis of differentially-expressed genes by EV71 infection. (**A**–**C**) The top 20 enriched pathways associated with differentially-expressed mRNAs, *cis*- and *trans*-acting target genes in EV71-infected RD cells, respectively. (**D**–**F**) The top 20 associated pathways in differentially-expressed mRNAs, *cis*- and *trans*- regulated mRNAs in EV71-infected skeletal muscle of mice, respectively.

To further explore the functions of these DEGs upon EV71 infection, the differentially regulated protein-coding RNAs and mRNAs were selected to conduct the GO enrichment and KEGG pathway analysis. Considering *p* value and rich factor, the top 10 significant enrichment GO terms of molecular function, biological process and cellular component aspects are shown in Figure 6 and Supplementary Tables S6 and S7. Our results indicate that the most enriched GO terms in these three aspects are cell division (ontology: biological process, GO: 0051301), protein binding (ontology: molecular function, GO: 0005515) and nucleus (ontology: cellular component, GO: 0005635) in RD cells, while in mice skeletal muscle, the GO terms in these three aspects are transport (ontology: biological process, GO: 0008610), protein binding (ontology: molecular function, GO: 0005515) and membrane (ontology: cellular component, GO: 0016020). Furthermore, the KEGG pathways analysis show that the identified mRNAs are mainly those that participate in protein processing in endoplasmic reticulum (hsa04141), pathways in cancer (hsa05200), human T cell lymphotropic virus type-1 (HTLV-I) infection (hsa05166), metabolic pathways (mmu01100), herpes simplex infection (mmu05168) and the mitogen-activated protein kinase (MAPK) signaling pathway (mmu04010). The top 20 enrichment pathways are listed in Figure 7 and Supplementary Tables S8 and S9.

### 3.6. qPCR Validation

**Figure 8:**
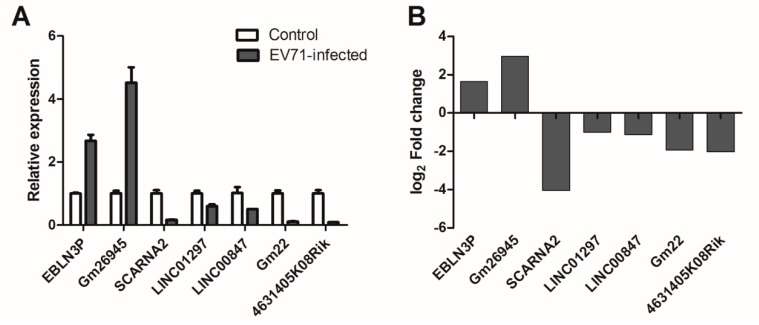
Comparisons of qPCR with RNA-seq results. (**A**) The validation results of qPCR. (**B**) The primary results of RNA-seq.

To verify the reliability of the RNA-seq results, we performed qPCR to detect the expression of lncRNAs in human RD cells and skeletal muscle of mice infected by EV71. Seven differentially-expressed lncRNAs were randomly selected with three biological duplicates for qPCR. The 2^−ΔΔCT^ method was used for calculating fold change in expression relative to controls. As shown in Figure 8A, these results indicated that the lncRNAs EBLN3P, Gm26945 were upregulated, and SCARNA2, LINC01297, LINC00847, Gm22 and 4631405K08Rik were downregulated after EV71 infection, which was consistent with the RNA-seq data (Figure 8B).

## 4. Discussion

EV71 is a common virus for severe HFMD in children and adults, and is considered as the emerging neurotropic enteroviral pathogen with *Enterovirus D68* (EV-D68), since poliovirus has been nearly eradicated [20,21]. To understand the interactions between EV71 and host, an appropriate experimental model is particularly important. In this study, human RD cells and Balb/c mice were selected as experimental models. Human RD cells (derived from rhabdomyosarcoma), are highly susceptible to EV71 and are often used to isolate EV71 from clinical specimens. Previous studies have shown that mouse skeletal muscle can act as a host organ for viral replication, which may play an important role in EV71 infection [22]. Our studies suggest that three-day-old Balb/c mice with i.p. inoculation of the ZZ1350 strain of EV71 exhibit high viral titer and pathological alterations in skeletal muscle at 7 dpi (Figure 2C,D).

Although a large number of studies have investigated the pathogenesis and virulence factors of EV71 [21], the molecular mechanism of EV71 infection remains largely unclear. In the past, studies mainly focused on the effect of EV71 infection on the regulatory function of coding proteins [23]. Recently, non-coding RNA including miRNAs and lncRNAs have also been demonstrated to play an important role in host and EV71 interaction [24]. A recent study showed that hsa-miR-23b could inhibit host EV71 replication through downregulation of the EV71 VP1 protein [25]. However, prior to the present study the regulation roles of lncRNA in EV71 infection-induced pathogenesis were rarely reported [26,27]. It was previously reported that results from microarray-based transcriptional assays are less accurate and not fully capable of predicting novel lncRNAs [28]. Yin et al. performed lncRNA and mRNA profiles in EV71-infected RD cells through microarray and identified 22,971 lncRNAs and 18,194 mRNAs from mock- and EV71-infected cells [26]. To the best of our knowledge, our study is the first of its kind to utilize the second generation of high throughput sequencing techniques to perform a quantitative and comprehensive analysis of lncRNAs combined with mRNAs both in EV71-infected human RD cells and skeletal muscle of mice. As a result, 24,517 lncRNAs including 2216 newly predicted lncRNAs and 143,640 mRNAs were identified in RD cells, and 11,087 lncRNAs including 1585 newly predicted lncRNAs and 60,469 mRNAs were obtained in mice skeletal muscle after EV71 infection, which enriched the information for further study on lncRNAs. Thus, these data provide novel insight into the unknown functions of lncRNAs in the process of EV71 infection.

As reported, viral infection can alter the expression profiles of lncRNAs [29]. Liu et al. performed RNA-seq in Huh7.5.1 cells with or without interferon (IFN)-α treatment to explore the regulation of lncRNAs in the hepatitis C virus (HCV) infection-induced type I IFN response. They obtained 7901 lncRNAs and identified 1062 host-encoded lncRNAs that were differentially expressed after IFN-α treatment [30]. In the present study, a total of 23 lncRNAs and 372 protein-coding RNAs were differentially expressed at significant levels (FC≥2 and p<0.05) between infected and uninfected RD cells. Interestingly, we also carried out in vivo experiments, which investigated the expression patterns of lncRNAs and mRNAs in response to EV71 infection in the skeletal muscle of mice. In skeletal muscle, the expression profiles of 104 lncRNAs and 2647 coding RNAs were significantly changed. Seven differentially-expressed lncRNAs identified in our studies were also selected randomly and verified using qPCR, and these qPCR results were consistent with the RNA-seq data to a large extent. Consequently, sequence homology analysis was carried out by aligning DEGs, but the results exhibited low homology in humans and mice (Supplementary Table S10). A previous study indicated that lncRNAs are usually expressed at lower levels than transcripts for protein-coding genes, and are highly specific to different tissues, species and developmental stages, which supports our results [31]. Many studies have shown that the expression features of lncRNAs are closely related to the development of diseases [11,12,13]. According to such differential expression patterns, in different diseases, lncRNAs can also be applied as biomarkers for the identification of diseases in the early stage of development [32,33]. As for our results, the most significantly upregulated and downregulated lncRNAs, respectively, were RP1-261G23.7 (FC=6.87) and SCARNA2 (FC=−16.45) in EV71-infected RD cells compared to that in controls (Supplementary Table S2). Nowadays, lncRNA studies are still preliminary and the functions of most lncRNA transcripts remain unknown. RP1-261G23.7 and SCARNA2 have not been reported yet; further gene knockout or overexpression studies are needed to verify the functions of DEGs during EV71 infection.

Accumulating evidence revealed that lncRNAs play a crucial role in regulating gene expression by interacting with DNA, protein-coding RNAs, or transcriptional regulatory protein molecules [13]. Functional analysis on the differentially-expressed mRNA and *cis*- and *trans*-acting target coding RNAs of differentially-expressed lncRNAs may help us understand the pathogenic mechanism of lncRNAs in response to EV71 infection. Yin et al. performed functional enrichment analysis on lncRNAs and nearby mRNA pairs in EV71-infected RD cells, and the results showed that the mRNAs were mainly found in alternative splicing, splice variants, phosphoproteins, the nucleus and they cytosol [26]. However, in this study, we found that the most significant functional group consisted of annotation terms found in protein binding, the nucleus, the cytosol and extracellular exosomes in EV71-infected RD cells (Figure 6). The reason for the inconsistency between the previous study and the present study may be that the previous study defined the *cis*-regulated target mRNAs located within 300 kb upstream and downstream of lncRNAs, but this study was only 100 kb. Our result showed the mis-regulated gene expression pathways were mainly involved in viral infectious disease, signal transduction, and immune system and metabolism processes (Figure 7). For differentially-expressed mRNAs in vivo, the most enriched GO terms were consistent with in vitro experiments and the mis-regulated gene expression pathways were mainly related to metabolism, viral infectious diseases, the immune system, signal transduction, neurodegenerative diseases and cancer. In particular, most significant enrichment pathways from in vitro and in vivo experiments are identical. Among them, majority pathways were associated with immune diseases and viral infectious disease, such as allograft rejection, antigen processing and presentation, chemokine signaling pathway, graft-versus-host disease, influenza A, Epstein–Barr virus infection, herpes simplex infection and HTLV-I infection. These findings indicate that EV71 infection mainly alters the host immune response, infectious diseases, protein binding, cellular components and metabolism, as well as nervous system diseases, which provide a new, broad categorization of potential lncRNA functions during EV71 infection. EV71 infection is well known to suppress host innate immunity, by which fashion it can continue to replicate in host cells [21]. He et al. indicated that fibronectin (FN) binding to integrins can enhance EV71 infection, however, knockout of FN can significantly reduce viral load and depress viral binding to host cells [34]. In addition, it has been reported that differentially-expressed mRNAs induced by EV71 infection are associated with cell cycle, cytoskeleton, apoptosis mediators, protein translation and metabolic processes [35,36]. Our results are consistent with the above findings. As for the association of neurodegenerative diseases and cancer in the KEGG pathways analysis, we summarize two possible reasons. First, as shown in our previous study, neurodegenerative changes were observed in the brain and spinal cord of infected mice [37]. Second, the relative genes involved in neurodegenerative disorders and cancer development are pleiotropic, and their interactions are profound and difficult to predict.

It is worth noting that our current study identified some differentially-expressed lncRNAs proven to be associated with host immune responses. For example, we calculated the long non-coding RNA metastasis associated lung adenocarcinoma transcript 1 (MALAT1) was upregulated with a 3.32 fold change, while NEAT1 was 3.42 fold downregulated in EV71-infected RD cells (Supplementary Table S5). Jin et al. found that the expression of MALAT1 in peripheral blood mononuclear cells (PBMCs) was upregulated in human immunodeficiency virus (HIV) infected patients, while nuclear enriched abundant transcript 1 (NEAT1) was reduced in the plasma of HIV infected patients and the expression level was correlated with CD4 T-cell count [38]. A ribonuclear complex built around hexamethylene bisacetamide inducible 1 (HEXIM1) and NEAT1 was previously identified as a key nuclear regulator of DNA-mediated activation of innate immune responses [39]. Maternally expressed gene 3 (MEG3) has been reported to play an important role in cancer initiation, metastasis, progression and chemo-resistance; we noted that MEG3 was highly overexpressed (FC=9.67) in EV71-infected skeletal muscle of mice. In our future studies, we will use specific inhibitors or RNA silencing techniques to explore the role of MEG3 in EV71 infection.

Emerging evidence has largely elucidated the mechanisms by which lncRNAs function. The target-mimetic, sponge/decoy function on microRNAs was recently uncovered, which suggests that lncRNAs could act as microRNA sponges, thereby reducing the post-transcriptional regulation of microRNAs [40]. Keniry et al. showed that miR-675 was embedded within the first exon of H19 lncRNA, and the excision of miR-675 from H19 was dynamically regulated by the stress response RNA binding protein [41]. What subverts our current understanding and will require additional future studies is that lncRNAs can encode some special proteins, including micropeptides and polypeptides, that play specific important biological functions [42,43].

In summary, we first performed a comprehensive analysis of lncRNAs and mRNA expression profiles in vitro and in vivo following EV71 infection using RNA-seq. We screened out numerous newly-predicted and differentially-expressed lncRNAs, and conducted functional analysis of these lncRNAs. Our results suggest that lncRNAs may participate in EV71 infection-induced pathogenesis through regulating immune responses, protein binding, cellular component biogenesis and metabolism, which provide a deeper insight into the pathogenic mechanisms of EV71 infection. Future investigations will be required to discover additional specific biomarkers at the early stages of infection, and to identify novel, efficient treatment strategies for EV71 eradication.

## Figures and Tables

**Table 1:** Primers used for qPCR.

lncRNA Symbol	Chromosome Location	Primer Sequences (5′-3′)		Products (bp)
EBLN3P	9	Forward	GGACGCGGCCTCCTTACCTC	189
		Reverse	GGCACGCGCTAGTTCCACAG	189
Gm26945	12	Forward	GGTGGCTCATGTGGATCACTGATG	87
		Reverse	ACGATGGACCTCCTGAGTGACAG	87
SCARNA2	1	Forward	CTGTGGCGTCGCGTGTGAG	97
		Reverse	CGCACGCACTCGCCTAACAC	97
LINC01297	22	Forward	ACGTCTCCATCCAGGACTGAGG	141
		Reverse	AAGACTGCCATCCAAGGAAGCG	141
LINC00847	5	Forward	GGACTTGCCAGCCTTCAGAACTC	181
		Reverse	GGTGATTGGATAGTGCCTGCCTAC	181
Gm22	8	Forward	GCCACAGCCATCAGCACAGC	127
		Reverse	CGCAACACCACTGACATGGAGAG	127
4631405K08Rik	1	Forward	AGAGTCACTGTCCTCCGCTTATCC	199
		Reverse	GCTGGCTCTCCTCTTCAATGAGTG	199
β-actin		Forward	AGCGAGCATCCCCCAAAGTT	285
(*Homo sapiens*)		Reverse	GGGCACGAAGGCTCATCATT	285
β-actin		Forward	GTGACGTTGACATCCGTAAAG	287
(*Mus musculus*)		Reverse	GTAACAGTCCGCCTAGAAGCAC	287

## Supplementary Materials

The following are available online at http://www.mdpi.com/1999-4915/10/10/556/s1, Table S1: lncRNA CPC, CPAT, Pfam and CNCI predicted, Table S2: lncRNA expression level, Table S3: mRNA expression level, Table S4: cis-regulation target genes, Table S5: trans-regulation target genes, Table S6: GO annotation of DEGs in RD cell, Table S7: GO annotation of DEGs in mouse skeletal, Table S8: KEGG enrichment of DEGs in RD cell, Table S9: KEGG enrichment of DEGs in mouse skeletal, Table S10: sequence homology analysis
